# Treatment Strategy for Fatal Arrhythmias in Ebstein’s Anomaly Combined With Leadless Pacemaker and S-ICD Implantations

**DOI:** 10.1016/j.jaccas.2022.04.016

**Published:** 2022-11-03

**Authors:** Mitsuru Takami, Koji Fukuzawa, Kunihiko Kiuchi, Kensuke Matsumoto, Yu Izawa, Ken-ichi Hirata

**Affiliations:** a Section of Arrhythmia, Division of Cardiovascular Medicine, Department of Internal Medicine, Kobe University Graduate School of Medicine, Kobe, Japan; b Division of Cardiovascular Medicine, Department of Internal Medicine, Kobe University Graduate School of Medicine, Kobe, Japan

**Keywords:** cardiac surgery, catheter ablation, Ebstein’s anomaly, leadless pacemaker, S-ICD, ventricular tachycardia, ACHD, adult congenital heart disease, ECG, electrocardiogram, ICD, implantable cardioverter-defibrillator, RA, right atrium, RV, right ventricle, S-ICD, subcutaneous implantable cardioverter-defibrillator, SVC, superior vena cava, VF, ventricular fibrillation, VT, ventricular tachycardia

## Abstract

The management of heart rhythm disorders in patients with adult congenital heart disease and limited vascular access is challenging. We present the case of a 38-year-old woman with Ebstein’s anomaly who underwent implantation of a combination of a leadless pacemaker and a subcutaneous implantable cardioverter-defibrillator to manage fatal arrhythmias. (**Level of Difficulty: Intermediate.**)

## Central Illustration

**Figure:**
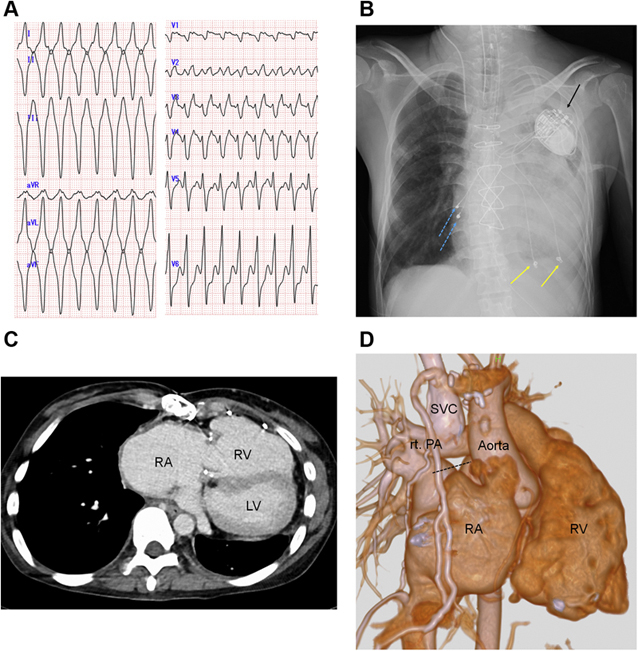

## History of Presentation

**Figure 1:**
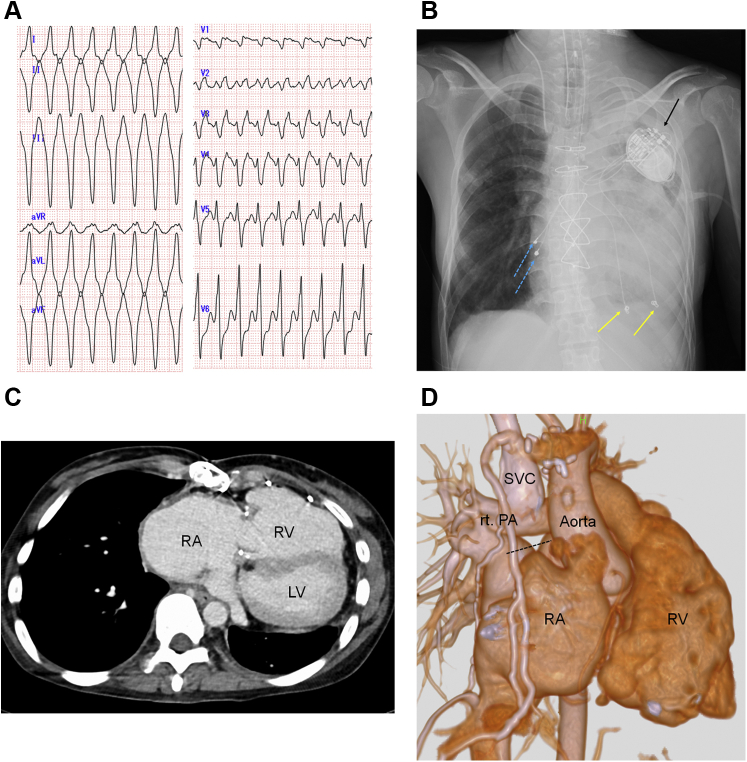
ECG, Radiography, and CT Imaging **(A)** Electrocardiogram (ECG) of the clinical ventricular tachycardia. **(B)** Chest radiograph. The **yellow arrows** indicate the epicardial ventricular leads. The **blue dotted lines** indicate the epicardial atrial leads. The **black arrow** indicates the pacemaker generator. **(C)** Computed tomography (CT) image showing significant enlargement of the right atrium (RA) and right ventricle (RV). **(D)** The 3-dimensional computed tomography image. The superior vena cava (SVC) is connected to the right pulmonary artery (rt. PA) by using the bidirectional Glenn procedure. The top of the right atrium is closed **(black dotted line).** LV = left ventricle.

A 38-year-old woman was referred to our hospital (Kobe University Hospital, Kobe, Japan) with palpitations and syncopal episodes. She had pallor and was hemodynamically unstable. Her blood pressure was 78/48 mm Hg, her pulse rate was 180 beats/min, and her peripheral oxygen saturation was 93% on room air. An electrocardiogram (ECG) revealed ventricular tachycardia (VT) (Figure 1A). Although she underwent electrical cardioversion, the VT recurred easily. She was sedated and intubated (Figure 1B), and then she was admitted to the intensive care unit.

**Learning Objectives**

• To recognize the risk of device interaction between the leadless pacemaker and S-ICD.
• To understand the importance of the order of the implantations to minimize any interaction.
• To understand the pacing mode during the screening test for S-ICD and VF induction.

## Past Medical History

She had Ebstein’s anomaly and a history of 3 cardiac surgical procedures:

1. At the age of 9 years, a tricuspid valve replacement was performed.
2. At the age of 18 years, a bidirectional Glenn procedure and plication of an enlarged right atrium (RA) and right ventricle (RV) were performed for ventricular unloading. The superior vena cava (SVC) was connected to the right pulmonary artery (Figures 1C and 1D).
3. Later, tricuspid valve degeneration, a perivalvular leak, atrial flutter, and sick sinus syndrome (intermittent sinus arrest) developed. At the age of 32 years, she underwent a repeat tricuspid valve replacement and a right atrial maze procedure. Unipolar pacemaker leads were implanted in the epicardium of the RA and RV (Figure 1B). Later, the atrial lead broke, and the ventricular epicardial lead exhibited high pacing thresholds (0.4 ms-5.0 V).

## Differential Diagnosis

Atrial tachycardia with aberrant conduction was considered in the differential diagnosis of wide QRS complex tachycardia.

## Management

On day 5 of admission, the patient underwent catheter ablation of the VT. Although low voltages and abnormal electrical potentials were widespread in the RV, the abnormal potentials were eliminated, and the VT did not recur.

The patient required implantation of a secondary preventive implantable cardioverter-defibrillator (ICD); however, there was no venous access to the RA from the SVC. There were 2 options:

1. Implantation of a transvenous ICD lead or shock coil on the epicardium by open chest surgery. Severe epicardial adhesions would be expected because of previous open chest surgical procedures. She also had chronic atelectasis of the lung; therefore, open chest surgery would be highly risky because of her impaired pulmonary function.
2. Implantation of a subcutaneous implantable cardioverter-defibrillator (S-ICD), in which case the electrical interaction between the S-ICD and the pacemaker would need to be noted. This is especially so for a pacemaker with unipolar pacing because fatal electrical crosstalk with an S-ICD has been reported.^1^ Considering the limited access to the RV, a leadless pacemaker (bipolar pacing) implantation would be the best option. After discussing the treatment strategy with the team of cardiac specialists, the health care team, and the ethics committee, the patient chose a combination therapy of a leadless pacemaker and an S-ICD.

**Figure 2:**
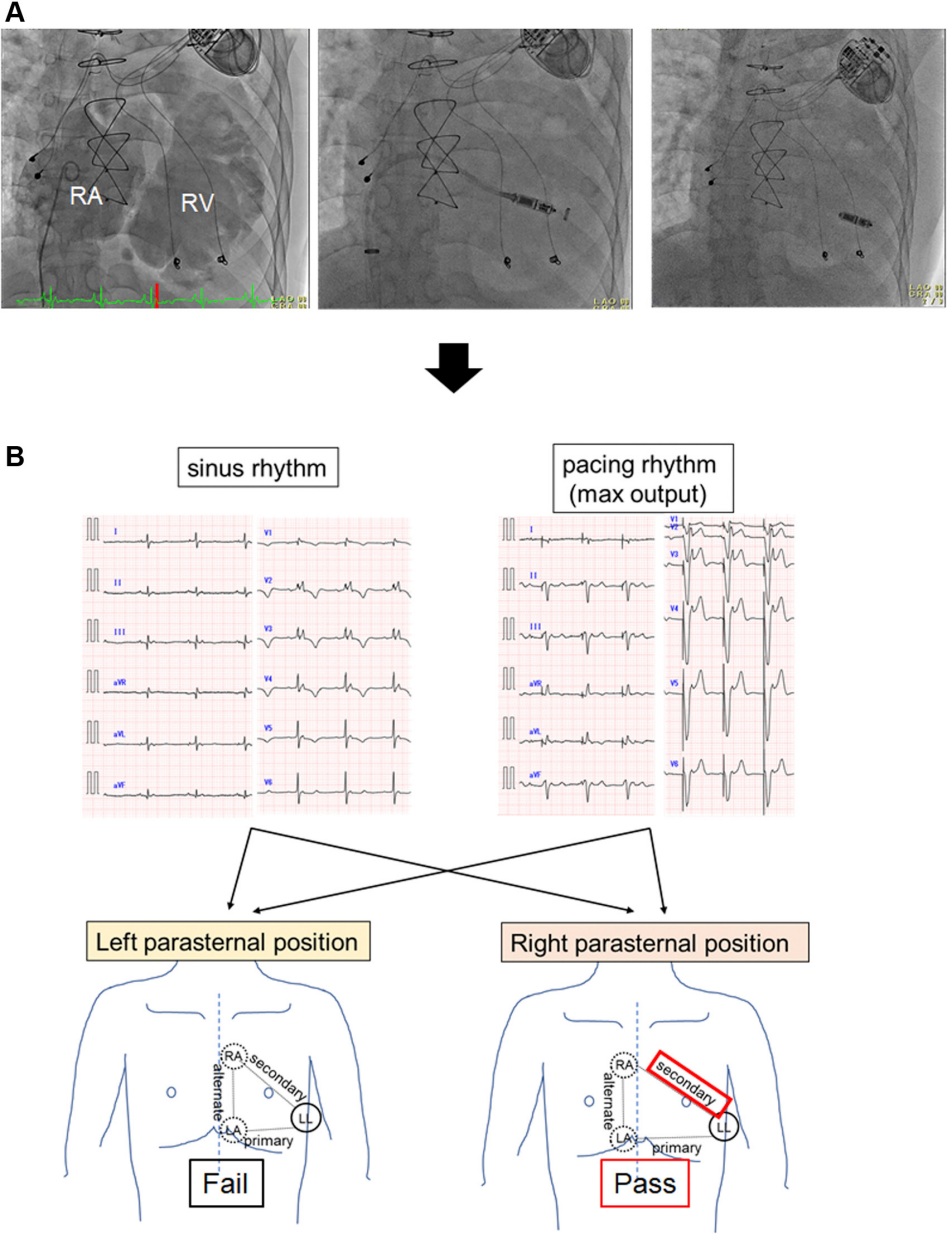
Leadless Pacemaker Implantation and the S-ICD Screening Test **(A)** On day 34, a leadless pacemaker was implanted. **(B)** On day 37, an electrocardiogram screening test for the subcutaneous implantable cardioverter-defibrillator (S-ICD) implantation was performed under the intrinsic rhythm and paced rhythm at the maximal output from the leadless pacemaker to rule out any double count of the paced rhythm. This patient did not pass the screening test from the left parasternal position and passed it from the secondary vector in the right parasternal position. LA = left arm; LL = left leg; RA = right arm; other abbreviations as in Figure 1.

On day 34 after admission, a leadless pacemaker (Micra VR Transcatheter Pacing System, Medtronic) was implanted (Figure 2A). The voltage map during VT ablation showed that the RV septum’s voltage was relatively higher than that of the RV free wall. On the basis of this information, we carefully placed the Micra pacing system in the lower septum. The first 3 deployments could not electrically capture the RV. During the fourth deployment on the RV septum, the pacing threshold was high (0.40 ms-4.0 V, 1.0 ms-2.88 V); however, we accepted the high pacing threshold because the pacing percentage was <10%.

On day 37, we performed surface ECG screening tests (automated and standard ECG-based screening tests) for the S-ICD with the patient in the supine and sitting positions (Figure 2B). To investigate the interaction between the leadless pacemaker and the S-ICD, a screening test was performed during pacing (pacing mode: VOO 80 ppm at maximum output) and intrinsic rhythms. The screening test with the patient in the standard left parasternal position failed because of the low amplitude of the QRS complex during intrinsic rhythm and the pacing spike sensing during pacing rhythm. We chose the secondary vector in the right parasternal and upper 1 cm of electrode position for the following reasons: 1) the amplitude of the QRS complex during the intrinsic rhythm was stable; and 2) the pacing spike was not seen, and the amplitude of the T-wave was low during the pacing rhythm.

**Figure 3:**
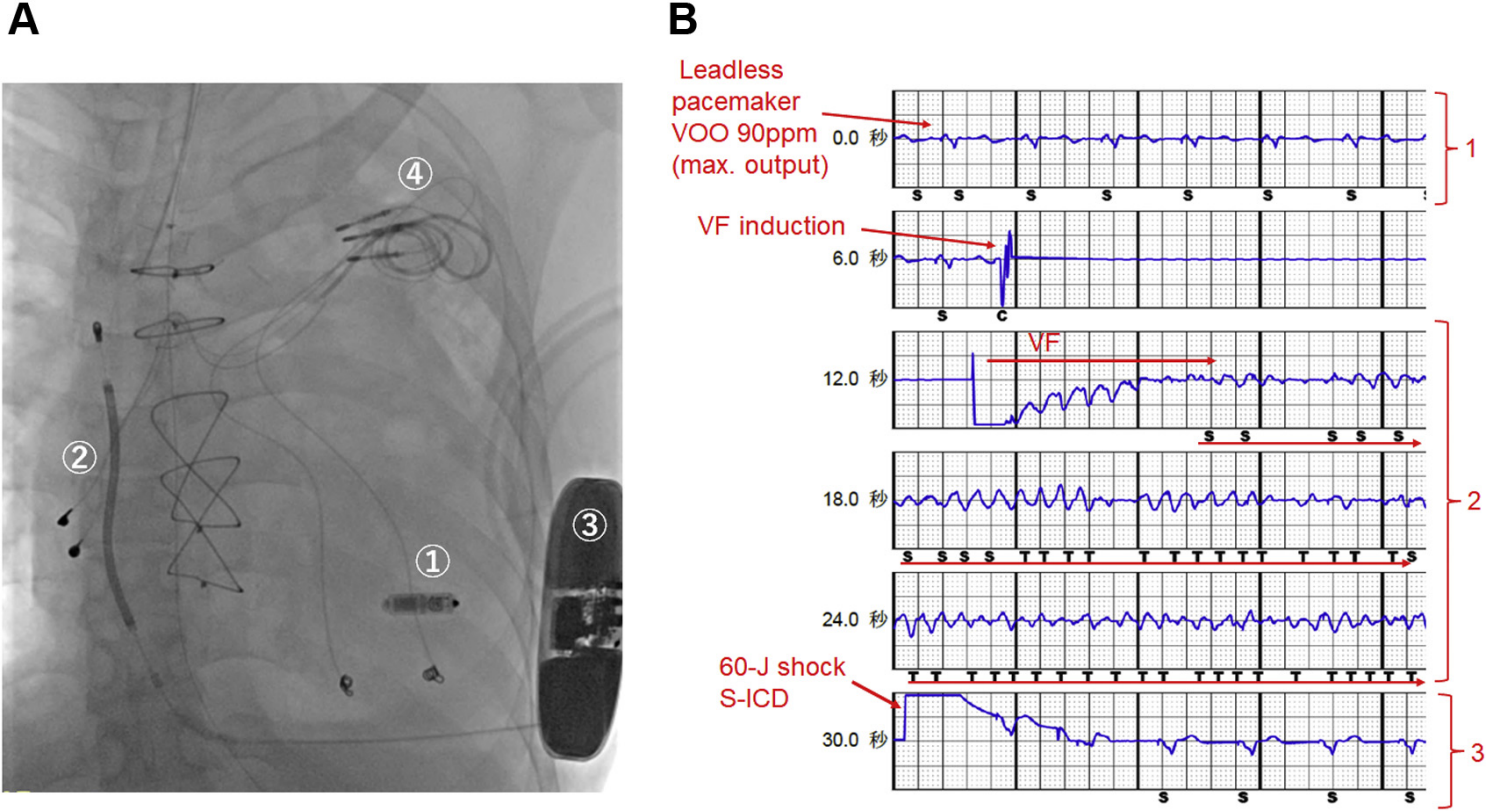
Implantation of S-ICD **(A)** Implantation of the subcutaneous implantable cardioverter-defibrillator (S-ICD): *1,* leadless pacemaker; *2,* the right parasternal position of the subcutaneous implantable cardioverter-defibrillator lead, *3,* subcutaneous implantable cardioverter-defibrillator generator; *4,* removal of the existing pacemaker. **(B)** Ventricular fibrillation (VF) induction test: *1,* ventricular fibrillation was induced during the paced rhythm (VOO) at maximal output from the leadless pacemaker; *2,* neither oversensing of the pacing spike nor undersensing of the ventricular fibrillation was observed; *3,* a 60-J shock from the subcutaneous implantable cardioverter-defibrillator terminated the ventricular fibrillation.

On day 44, an S-ICD (EMBLEM MRI S-ICD, Boston Scientific) was implanted (Figure 3A). A ventricular fibrillation (VF) induction test was performed under the VOO pacing mode at the maximum output from the leadless pacemaker (Figure 3B). Neither oversensing of the pacing spike nor undersensing of the VF was observed. A 60-J shock from the S-ICD successfully terminated the VF, and the leadless pacemaker exhibited no parameter changes. The existing pacemaker generator was then removed. The clinical VT rate was 180 beats/min. The patient also had atrial tachyarrhythmia; therefore, we set the S-ICD’s conditional shock zone to 170 to 200 beats/min and the shock zone to above 200 beats/min.

## Discussion

To our knowledge, this is the first successful case of adult congenital heart disease (ACHD) therapy with a combination of a leadless pacemaker and S-ICD implantation. The patient already had a conventional pacemaker with epicardial unipolar leads. According to the manufacturer of that device, a unipolar pacing system is a contraindication to S-ICD implantation for the following reasons: 1) S-ICD has a risk of oversensing the unipolar pacing spike (double counting), and this would cause inappropriate shocks; and 2) the pacing spike would affect the S-ICD sense amplifier during VT or VF, and this would cause undersensing of the VT or VF and failure of shock delivery.^1^ Therefore, we chose combination therapy with an implantation of a leadless pacemaker (bipolar pacing) and an S-ICD. However, the electrical interaction between devices could still be a concern. The combination therapy has been reported in older patients with vascular occlusion, post-infection.^2^, ^3^, ^4^ Most previous reports described an order of the procedure in which the S-ICD was implanted first, followed by the leadless pacemaker. However, in the present case, we first performed leadless pacemaker implantation, after which the patient underwent an S-ICD screening test, and then we implanted the S-ICD. The screening tests for both the intrinsic and pacing rhythms before implanting the S-ICD are significant to avoid double counting the paced QRS complex. Patients with ACHD exhibit the following characteristics: 1) complex cardiac anatomy; 2) myocardial injury and/or an abnormal conduction system because of repeated cardiac surgical procedures; and 3) a relatively younger age. It has also been reported that 17% of contemporary patients with ACHD are ineligible for an S-ICD screening test, especially patients with a wide QRS complex and/or those needing ventricular pacing.^5^ Therefore, we implanted the leadless pacemaker before the S-ICD. Ng et al^4^ reported the simultaneous implantation of a leadless pacemaker and an S-ICD. However, we preformed the device implantation procedures on separate days. Screening tests for S-ICDs should be performed with the patient in the 2 different positions (supine and standing positions) to investigate the changes in the vectors. If we performed simultaneous implantations, it would be difficult to perform the screening test with the patient in the standing and sitting positions.

It was recently reported that implanting another leadless pacemaker in a different location in the RV was suboptimal in patients with a coexistent S-ICD.^6^ Hence, in the present case, the implantation site for the future replacement of the leadless pacemaker will be carefully selected.

## Follow-Up

At the 18-month follow-up, no VT recurred, and no interaction between the leadless pacemaker and S-ICD was observed. The RV pacing threshold of the leadless pacemaker slightly improved (0.4 ms-3.6 V, 1.0 ms-2.75 V). The pacing percentage was 2% to 5%, and the estimated battery longevity was 7 to 9 years.

## Conclusions

This stepwise treatment strategy, combined with the implantation of a leadless pacemaker and an S-ICD, could manage bradyarrhythmias and tachyarrhythmias in patients with Ebstein’s anomaly. The order of the interventional procedure and the pacing mode during the screening test and the VF induction test are important to minimize the risk of electrical interactions.

## Funding Support and Author Disclosures

The Section of Arrhythmia, Division of Cardiovascular Medicine, Department of Internal Medicine, Kobe University Graduate School of Medicine is supported by an endowment from Medtronic Japan and Abbott Japan; Drs Takami and Fukuzawa have reported belonging to the section; and Dr Hirata has reported chairing the section. All other authors have reported that they have no relationships relevant to the contents of this paper to disclose.
